# Learning Domain-Independent Deep Representations by Mutual Information Minimization

**DOI:** 10.1155/2019/9414539

**Published:** 2019-06-16

**Authors:** Ke Wang, Jiayong Liu, Jing-Yan Wang

**Affiliations:** ^1^College of Mathematics, Sichuan University, Chengdu 610065, China; ^2^College of Cybersecurity, Sichuan University, Chengdu 610065, China; ^3^New York University Abu Dhabi, Abu Dhabi, UAE

## Abstract

Domain transfer learning aims to learn common data representations from a source domain and a target domain so that the source domain data can help the classification of the target domain. Conventional transfer representation learning imposes the distributions of source and target domain representations to be similar, which heavily relies on the characterization of the distributions of domains and the distribution matching criteria. In this paper, we proposed a novel framework for domain transfer representation learning. Our motive is to make the learned representations of data points independent from the domains which they belong to. In other words, from an optimal cross-domain representation of a data point, it is difficult to tell which domain it is from. In this way, the learned representations can be generalized to different domains. To measure the dependency between the representations and the corresponding domain which the data points belong to, we propose to use the mutual information between the representations and the domain-belonging indicators. By minimizing such mutual information, we learn the representations which are independent from domains. We build a classwise deep convolutional network model as a representation model and maximize the margin of each data point of the corresponding class, which is defined over the intraclass and interclass neighborhood. To learn the parameters of the model, we construct a unified minimization problem where the margins are maximized while the representation-domain mutual information is minimized. In this way, we learn representations which are not only discriminate but also independent from domains. An iterative algorithm based on the Adam optimization method is proposed to solve the minimization to learn the classwise deep model parameters and the cross-domain representations simultaneously. Extensive experiments over benchmark datasets show its effectiveness and advantage over existing domain transfer learning methods.

## 1. Introduction

### 1.1. Background

Transfer learning is a machine learning problem which deals with data from two domains [1–6]. One domain is target domain, and in this domain, we aim to learn an effective machine learning model for prediction. Another domain is source domain, in which we have sufficient labeled data points. Usually, in the target domain, the labeled data points are of a small number, which is not sufficient to learn an effective model. Thus domain transfer learning tries to transfer the knowledge in the source domain to the target domain to help the learning in the target domain. Although the target domain and source domain share the same input and output space, the distribution of the input data points of two domains is of significant difference. For example, in the problem of text topic categorization, the newspaper article is a source domain, where almost all the articles are labeled well, and the personal communication message is a target domain. Usually, the message texts are not labeled, or only a small number of them are labeled. It is natural to use the newspaper articles and the corresponding labels to help the learning of model for the categorization of the message texts. However, the newspaper articles are written normally, while the personal messages are usually written casually. Thus, the usage of words and the writing styles are very different. This leads to the significant difference between the distributions of the source domain (newspaper article) and the target domain (personal message). Transfer learning aims to build a predictive model for the target domain by utilizing the data points of both domains, even though they are of different distributions.

In this case, it is very necessary to map the data points of both domains to a common data space so that they lie in the same distribution, and we can directly train a model for the target domain by using both domains' data points' representations. Another solution is to learn a model for the source domain first and then adapt it to the target domain. In this paper, we focus on the first solution where the data points are mapped to a common space. This solution aims to learn domain transferable representations for data points in different domains. Different representation learning methods have been applied for domain transferable representation learning, including multikernel learning [7–10], deep learning [11–20], nonnegative matrix factorization [21–24], sparse coding [25–28], etc. For the domain distribution matching-based domain transfer learning, the most popular method is based on the maximum mean discrepancy criterion. It calculates the means of the representations of data points of source and target domain and minimizes the squared *ℓ*_2_ norm distance to match the two domains.

In this paper, we study the problem of learning domain transfer representations. However, we do not consider the distribution matching of two domains but consider learning representations which can be directly generalized to two domains.

### 1.2. Related Works

In this section, we briefly introduce the state-of-the-art methods for transferable representation learning.

  Theauthors in [4] present a novel method to learn deep networks for domain adaptation. The proposed method maps the outputs of all layers of deep networks to reproduce kernel Hilbert spaces and tries to match the distributions of these layers' outputs of target and source domains. Moreover, the kernel space mapping is conducted by applying multikernel learning, where the optimal kernel function is a weighted linear combination of multiple kernels. This is different from the conventional transfer learning methods, which only matches the distributions of outputs of the last layer of source and target domains. The mismatching of the two distributions is measured by the maximum mean discrepancy criterion, which actually minimizes the squared Euclidean distance between the mean outputs of the source and target domains of the corresponding layer.
  The authors in [6] proposed to learn a domain transfer learning model for feature space independent domains. In this case, the source domain and target domain have completely different feature space. The source domain data points are mapped to target domain data points. The mapping guarantees that any source domain data point is mapped to a target domain point with the same class label. The target domain and source domain (mapped to target domain) are both represented by kernel matrices. To measure how well the two domains are allied, the Hilbert Schmidt Independence Criterion is applied. It calculates the trace of the product of the target domain kernel matrix and the mapped source domain kernel matrix. By maximizing the trace of the product of target and source, the distributions of the source and target domains are aligned and matched.
  The authors in [1] proposed a novel method for transfer learning. It selects the data points from the source domain for the target domain learning problem. To be specific, it assigns a weight to each source domain data point, which plays the role of selective weight. This weight has two functions. The first function is to select important source domain data points to represent the source domain to match the target domain. Instead of calculating the mean vector of the source domain features, this method calculates the weighted mean and matches it to the target domain by the maximum mean discrepancy criterion. The second function is to weight the loss function of the target domains. The target domain data points' weights and the classifier parameters are also learned simultaneously in an iterative algorithm.
  The authors in [2] developed a novel multikernel classifier for domain transfer learning. It constructs the kernel function by multikernel combination with learned weights. Meanwhile, the kernel weights and classifier parameters are learned simultaneously. To match the source domain and target domain, the data points of two domains are mapped to a nonlinear Hilbert space, and their distributions are matched in this space. The learning algorithm minimizes the classification losses over the labeled data points of both domains and the squared Euclidean distance between the mean multikernel representations of tow domains under the maximum mean discrepancy criterion.

### 1.3. Our Contributions

#### 1.3.1. Motive

All the above methods are based on the matching of two domains' distributions of the data representations. The two key components of this method are the representation of distributions and the metric of the mismatching of the two distributions. In this paper, we give up this framework and propose a completely different framework for domain transfer learning. We observed that for an ideal representation model across two different domains, from its output of one data point, we cannot tell which domain it is from. At the same time, we can separate it from its true class and the other classes according to its output of the cross-domain representation model. It means that the representation of a data point is independent of its domain, but closely relevant to its class. Thus, instead of measuring the mismatch of source and target domain distributions, we measure the independence of the representations and domain-belonging indicators of the data points. To measure the dependency between the representation and the domain indicator, we employ the mutual information. By minimizing the mutual information between them, we learn the domain-independent representations. Meanwhile, we also propose to maximize the margin of each data point so that it can be separated from data points from other classes and kept close to the data points from the same classes.

#### 1.3.2. Our Method

Motivated by the above ideas, we propose a novel deep learning model for the representation of data points of transfer learning problems. Firstly, to enhance the ability to discriminate data points of different classes, we propose to learn a unique deep convolutional network for each class, named classwise convolutional representation model. This is different from traditional domain transfer representation models, which learns a common model for all classes. To make the outputs of this model independent from the domain indicators, we propose to minimize the mutual information between the representation model outputs and the domain indicators. The mutual information estimation is based on the probability of representations and conditional probability of domain indicators given representations. We develop novel estimators for the conditional probability of domain indicators given representations. The estimator is defined over the neighborhood of the data point of the given representation, and it calculates the normalized summation of the soft weights of the data points from the input domain. To make the outputs of the model to be discriminate, we proposed to maximize the margin of each data point in the corresponding class. The margin is defined as the difference between intraclass dissimilarity and the interclass dissimilarity. The intraclass dissimilarity is defined in an intraclass neighborhood which contains a set of neighboring data points from the same class, while the interclass dissimilarity is defined in an interclass neighborhood which contains a set of neighboring data points from the other classes. To learn the representation model parameters, we build a unified learning framework. The objective function is defined by combining the margins, mutual information, and a squared *ℓ*_2_ norm term to control the complexity of the model. An iterative algorithm based on Adam is developed to solve the problem.

**Remark 1 .**

**Figure 1:**
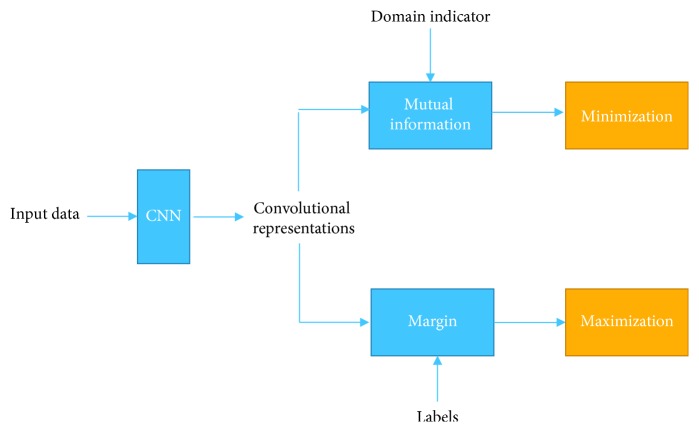
Overall diagram of the proposed learning framework for minimum mutual information domain transfer representation.

The overall diagram of the proposed learning framework for each class is given in Figure 1. As we can see from the figure, for each CNN model, its outputs are regularized by two types of auxiliary information: the domain indicator and the class label. Our framework calculates the mutual information between the CNN representations and the indicators of domains and minimizes it. Meanwhile, it calculates the margin from class label and maximizes it. In this way, this framework enables the CNN model to be discriminative and insensitive to the diversities of domains.

Our contributions are of three folds:

1. For the first time, the idea of learning cross-domain representations which are independent of domains is proposed for transfer learning. Instead of learning representations and making the distributions of two domains' representations to match each other, we directly learn representations which are independent of their domain-belonging indicators. The mutual information is used to measure such dependency of representations and domain indicators, and it is minimized to seek the domain-independent representations.
2. We develop a novel and practical representation learning method to minimize the mutual information between the data points' representations and domain indicators. The mutual information between representations and domain indicators of data points is estimated according to the probability of representation and the conditional probability of domain indicator given representation. We estimate the conditional probability of a data point's domain indicator given its representation over its neighborhood. It is calculated as a summation of the normalized Gaussian kernel based similarity measured of the data points in the neighborhood but from the considering domain.
3. We propose a novel transfer learning framework for learning domain transfer deep representation models. It is a classwise model and we learn the parameters by simultaneously maximizing the margin of each data point of this class and minimizing the mutual information between the data points' representations and their domain indicators. An iterative algorithm is developed to learn the optimal representations and the parameters of the model to output these representations.

### 1.4. Paper Organization

The paper is organized as follows: in Section 2, we introduce the proposed method in detail, including its mathematical modeling, problem optimization, and iterative algorithm design. In Section 3, we evaluate the proposed method over several transfer learning benchmark datasets to compare it against state-of-the-art transfer learning methods. In Section 4, we give the conclusion of this paper and some future works.

## 2. Methods

### 2.1. Definition of Symbols

In this section, we give a list of detailed definitions of the symbols used in the following sections.

### 2.2. Problem Modeling

We assume we have a set of *n* training data points, denoted as *𝒳*={*X*_1_,…, *X*_*n*_}, where *X*_*i*_={**x**_*i*1_,…, **x**_*in*_*i*__} is the *i*-th data point, which is composed of *n*_*i*_ instances, and **x**_*ij*_ is the *j*-th instance of the *i*-th data point. For the computer vision problem, a data point is an image, and an instance is an image patch, while for the natural language process problem, a data point is a sentence, and an instance is the embedding vector of a word.

#### 2.2.1. Large Margin Class-Specific Convolutional Representations

We consider a classification problem of *L* classes, the training set can be divided into subsets of *L* classes and a set of unlabeled data points whose class labels are not known yet. The training set can be denoted as follows:

(1) $$\begin{array}{c} X=X_{1}\;\cup \;\cdots \;\cup \;X_{L}\;\cup \;X_{U}, \end{array}$$

where *𝒳*_*l*_ is the subset of the *l*-th class and *𝒳*_*U*_ is the subset of the unlabeled data points.

For the *l*-th class, we learn a class-specific deep CNN model to represent the data point *X*,

(2) $$\begin{array}{c} f_{l}:X⟶z=f_{l}\left(X;\;W_{l}\right)\in {\mathbb{R}}^{m}, \end{array}$$

which outputs a vector of *m* dimensions as the class-specific convolutional vector, and *W*_*l*_ presents the parameters of the model.

**Remark 2 .**

We choose to learn the convolutional representations due to the following two reasons:

1. CNN model is good at extracting local patterns by utilizing a large number of sliding local filters, while in most domain transfer applications discussed in this paper, the local patterns play the most important role. For example, in the cross-domain image categorization task, for two images of different domains but containing the same object, the CNN model can capture the local region of the object with some local filters while ignoring the contexts which may vary in different domains. Another example is the text-related tasks; long sentences of the same topic may have different linguistic styles of different domains, but still contains short phrase, which could be captured by the CNN model effectively by its sliding local filters to extract features from short phrases.
2. CNN model, compared to the other deep learning models, such as recurrent neural network (RNN), has a more efficient training process. The CNN model has a parallel structure, and the responses of a sliding filter are calculated independently from each other; thus, its computing can be easily paralleled by GPU. This is different from the RNN model which has a sequential structure, where the response of a node is calculated based on the response of the previous nodes, which makes its computing time longer than the CNN model.

Naturally, we hope the class-specific convolutional representations can separate the data points of the *l*-th class and the other classes as far as possible so that the classification performance can be improved. To this end, we propose to learn to discriminate convolutional representations for the data points of *l*-th class, by maximizing the local margin of each data point in this class. For the local margin of a data point in the *l*-th class, *X*_*i*_ ∈ *𝒳*_*l*_ is defined by its intraclass neighborhood and interclass neighborhood. The intraclass neighborhood is the set of *κ* nearest neighboring data points in the same class, *𝒳*_*l*_:

(3) $$\begin{array}{c} N_{i}^{+}=\kappa -\arg\;\;\min_{X_{j}:X_{j}\in X_{l}}{\left\|z_{i}-z_{j}\right\|}_{F}^{2}, \end{array}$$

where the *ℓ*_2_ norm distance between their *l*-th class-specific convolutional representations is used to measure the distance of neighbors. Meanwhile, the interclass neighborhood is the set of *κ* nearest neighboring data points from a different class:

(4) $$\begin{array}{c} N_{i}^{-}=\kappa -\arg\;\;\min_{X_{j^{\prime }}:X_{j^{\prime }}\in X_{l^{\prime },l^{\prime }\neq l}}\;\;{\left\|z_{i}-z_{j^{\prime }}\right\|}_{F}^{2}. \end{array}$$

Note that to search for the interclass neighbors of *X*_*i*_, we use the convolutional representations of its class, the *l*-th class, even for the data points of the other classes. We further calculate an affinity measure for *X*_*i*_ and a data point *X*_*j*_ from *𝒩*_*i*_^+^, according to their class-specific convolutional representations and a Gaussian kernel function:

(5) $$\begin{array}{c} A_{ij}^{+}=\frac{g\left(z_{i}-z_{j}\right)}{\sum_{X_{j^{\prime }}\in N_{i}^{+}}g\left(z_{i}-z_{j^{\prime }}\right)}, \end{array}$$

where *g*(*x*)=exp(−‖*x*‖_*F*_^2^/2*σ*^2^).

Similarly, we also calculate the interclass affinity between *X*_*i*_ and data points in *𝒩*_*i*_^−^:

(6) $$\begin{array}{c} A_{ij^{\prime }}^{-}=\frac{g\left(z_{i}-z_{j^{\prime }}\right)}{\sum_{X_{j^{\prime }}\in N_{i}^{-}}g\left(z_{i}-z_{j^{\prime }}\right)}. \end{array}$$

The local margin of *X*_*i*_ is defined as the difference between the weighted intraclass convolutional dissimilarity and the interclass dissimilarity:

(7) $$\begin{array}{c} m_{l}\left(X_{i}\right)=\underset{X_{j}\in N_{i}^{-}}{\sum }A_{ij^{\prime }}^{-}{\left\|z_{i}-z_{j^{\prime }}\right\|}_{F}^{2}-\underset{X_{j}\in N_{i}^{+}}{\sum }A_{ij^{\prime }}^{+}{\left\|z_{i}-z_{j}\right\|}_{F}^{2}. \end{array}$$

We proposed to maximize the local margin to improve the ability to separate data points of the *l*-th class from the other classes. To this end, we minimize the following objective function of margins over the data points of the *l*-th class to learn the convolutional representation network parameters:

(8) $$\begin{array}{c} \underset{W_{l}}{\min}\underset{X_{i}\in X_{l}}{\sum }\left(\underset{X_{j}\in N_{i}^{+}}{\sum }A_{ij^{\prime }}^{+}{\left\|z_{i}-z_{j}\right\|}_{F}^{2}-\underset{X_{j}\in N_{i}^{-}}{\sum }A_{ij^{\prime }}^{-}{\left\|z_{i}-z_{j^{\prime }}\right\|}_{F}^{2}\right). \end{array}$$

#### 2.2.2. Minimum Mutual Information Domain Adaptation

Since we are considering the problem of domain transfer learning, the training data points are from a source domain and a target domain, and we denote the source domain training set as *𝒳*_*S*_ and *𝒳*_*T*_; thus, *𝒳*=*𝒳*_*S*_  ∪  *𝒳*_*T*_. We introduce a domain indicator for each data point *X*_*i*_ to present which domain it is from, *π*_*i*_ ∈ {source, target}, where *π*_*i*_=source indicates that *X*_*i*_ is a source domain data point, while *π*_*i*_=target indicates that it is a target domain data point. Naturally, we hope the classwise convolutional representations of the source and target domains are mapped to a common space of the same distribution. To this end, we impose that the representations of the data points and their domain indicators are independent of each other so that from the presentation, we cannot measure which domain it is from. To measure the mutual dependence between the classwise representation **z** and the domain indicator *π*, we proposed to use the mutual information between them, *I*(*π*; **z**).

**Remark 3 .**

According to the probability theory and information theory, the mutual information between two variables is a measure of the mutual dependence between them. For two variables, *x*, and *y*, the definition of mutual information of *x* and *y* is calculated by the double integral as follows:

(9) $$\begin{array}{c} I\left(x;y\right)=\int_{y}\int_{x}p\left(x,y\right)\log\left(\frac{p\left(x,y\right)}{p\left(x\right)p\left(y\right)}\right)dx\;dy, \end{array}$$

where *p*(*x*, *y*) is the joint probability function of *x* and *y* and *p*(*x*)(*p*(*y*)) is the probability function of *x*(*y*). For the discrete variables, the mutual information is calculated by the double sum:

(10) $$\begin{array}{c} I\left(x;y\right)=\underset{y}{\sum }\underset{x}{\sum }p\left(x,y\right)\log\left(\frac{p\left(x,y\right)}{p\left(x\right)p\left(y\right)}\right). \end{array}$$

According to the mutual information's relation to Kullback–Leibler divergence,

(11) $$\begin{array}{c} I\left(x;y\right)=\underset{y}{\sum }p\left(y\right)D_{\text{KL}}\left(p\left(x\;|\;y\right)p\left(x\right)\right)\\ =\underset{y}{\sum }p\left(y\right)\underset{x}{\sum }p\left(x\;|\;y\right)\text{log}\left(\frac{p\left(x\;|\;y\right)}{p\left(x\right)}\right), \end{array}$$

where *D*_KL_(*p*(*x* | *y*)*p*(*x*)) is the Kullback–Leibler divergence between *p*(*x* | *y*) and *p*(*x*) and *p*(*x* | *y*) is the conditional probability of *x* given *y*. Following equation (11), the mutual information between *π* and **z** is defined as follows:

(12) $$\begin{array}{c} I\left(\pi ;z\right)=\underset{z}{\sum }p\left(z\right)\underset{\pi }{\sum }p\left(\pi \;|\;z\right)\text{log}\left(\frac{p\left(\pi \;|\;z\right)}{p\left(z\right)}\right). \end{array}$$

To estimate the mutual information over the training set, we propose to recalculate *I*(*π*; **z**) as follows:

(13) $$\begin{array}{c} I\left(\pi ;z\right)=\underset{i:X_{i}\in X}{\sum }p\left(z_{i}\right)\underset{\pi \in \left\{\text{source},\text{target}\right\}}{\sum }p\left(\pi \;|\;z_{i}\right)\text{log}\left(\frac{p\left(\pi \;|\;z_{i}\right)}{p\left(z_{i}\right)}\right). \end{array}$$

In the following, we discuss how to estimate the conditional probability of domain indicator given the convolutional representation, *p*(*π* | **z**_*i*_), and the probability of the convolutional representation, *p*(**z**_*i*_).

#### 2.2.3. Estimation of *p*(*π* | **z**_*i*_)

To estimate the probability of *π* given a data point, we propose to calculate the density of *π* over the neighborhood of *X*_*i*_. *𝒳*_*i*_ is the set of *k*-nearest neighbors,

(14) $$\begin{array}{c} N_{i}=k-\arg\;\;\min_{X_{j}\in X}{\left\|z_{i}-z_{j}\right\|}_{F}^{2}, \end{array}$$

and the probability of *π* over *𝒳*_*i*_ is calculated as the empirical distribution,

(15) $$\begin{array}{c} p\left(\pi \;|\;z_{i}\right)=\frac{1}{\left|N_{i}\right|}\underset{X_{j}\in N_{i}}{\sum }\delta \left(\pi_{j}=\pi \right), \end{array}$$

where *δ*(*x*)=1 if *x* is true, otherwise 0. According to equation (15), *p*(*π* | **z**_*i*_) is the weighted summation of *δ*(*π*_*j*_=*π*) over *𝒩*_*i*_, and the weights are hard weight (1/|*𝒩*_*i*_|). We release the calculation of the weights as soft weight according to Gibbs distribution as follows:

(16) $$\begin{array}{c} p\left(\pi \;|\;z_{i}\right)=\underset{j:X_{j}\in N_{i}}{\sum }\omega_{ij}\delta \left(\pi_{j}=\pi \right),\\ \text{where }\omega_{ij}=\frac{g\left(z_{i}-z_{j}\right)}{\sum_{j^{\prime }\in N_{i}}g\left(z_{i}-z_{j^{\prime }}\right)}. \end{array}$$

The weights satisfy the constraints of ∑_*j*∈*𝒩*_*i*__*ω*_*ij*_=1 and *ω*_*ij*_ ≥ 0.

#### 2.2.4. Estimation of *p*(**z**_*i*_)

We assume the convolutional representations are evenly distributed; thus, we use a simple empirical distribution function to calculate the probability of **z**_*i*_ as follows:

(17) $$\begin{array}{c} p\left(z_{i}\right)=\frac{1}{n}. \end{array}$$

Substituting equations (17) and (15) in (13), we rewrite the mutual information between variables **z** and *π* as follows:

(18) $$\begin{array}{c} I\left(\pi ;z\right)=\frac{1}{n}\underset{i:X_{i}\in X}{\sum }\underset{\pi \in \left\{\text{source,target}\right\}}{\sum }\left(\underset{j:X_{j}\in N_{i}}{\sum }\omega_{ij}\delta \left(\pi_{j}=\pi \right)\right)\left(\log\left(\underset{j:X_{j}\in N_{i}}{\sum }\omega_{ij}\delta \left(\pi_{j}=\pi \right)\right)+\log\left(n\right)\right). \end{array}$$

To simplify the equations, we introduce the following variables:

(19) $$\begin{array}{c} h_{i}=\underset{j:X_{j}\in N_{i}}{\sum }g\left(z_{i}-z_{j}\right),\\ h_{i}^{s}=\underset{j:X_{j}\in N_{i},\pi_{j}=\text{source}}{\sum }g\left(z_{i}-z_{j}\right),\\ h_{i}^{t}=\underset{j:X_{j}\in N_{i},\pi_{j}=\text{target}}{\sum }g\left(z_{i}-z_{j}\right), \end{array}$$

so that

(20) $$\begin{array}{c} h_{i}=h_{i}^{s}+h_{i}^{t}\\ \underset{j:X_{j}\in N_{i}}{\sum }\omega_{ij}\delta \left(\pi_{j}=\text{source}\right)=\frac{h_{i}^{s}}{h_{i}},\\ \underset{j:X_{j}\in N_{i}}{\sum }\omega_{ij}\delta \left(\pi_{j}=\text{target}\right)=\frac{h_{i}^{s}}{h_{i}}. \end{array}$$

We rewrite equation (18) with *h*_*i*_, *h*_*i*_^*s*^, and *h*_*i*_^*t*^ as follows:

(21) $$\begin{array}{c} I\left(\pi ;z\right)=\frac{1}{n}\underset{i:X_{i}\in X}{\sum }\left[\frac{h_{i}^{s}}{h_{i}}\log\left(\frac{h_{i}^{s}}{h_{i}}\right)+\frac{h_{i}^{s}}{h_{i}}\log\left(n\right)+\frac{h_{i}^{s}}{h_{i}}\log\left(\frac{h_{i}^{s}}{h_{i}}\right)+\frac{h_{i}^{s}}{h_{i}}\log\left(n\right)\right]\\ =\frac{1}{n}\underset{i:X_{i}\in X}{\sum }\left[\frac{h_{i}^{s}\log\left(h_{i}^{s}\right)+h_{i}^{t}\log\left(h_{i}^{t}\right)}{h_{i}}+\log\left(n\right)-\log\left(h_{i}\right)\right]\\ =\frac{1}{nh_{i}}\underset{i:X_{i}\in X}{\sum }\left(\underset{j:X_{j}\in N_{i},\pi_{j}=\text{source}}{\sum }g\left(z_{i}-z_{j}\right)\right)\text{log}\left(\underset{j:X_{j}\in N_{i},\pi_{j}=\text{source}}{\sum }g\left(z_{i}-z_{j}\right)\right)+\frac{1}{nh_{i}}\underset{i:X_{i}\in X}{\sum }\left(\underset{j:X_{j}\in N_{i},\pi_{j}=\text{target}}{\sum }g\left(z_{i}-z_{j}\right)\right)\text{log}\left(\underset{j:X_{j}\in N_{i},\pi_{j}=\text{target}}{\sum }g\left(z_{i}-z_{j}\right)\right)-\frac{1}{n}\underset{i:X_{i}\in X}{\sum }\text{log}\left(\underset{j:X_{j}\in N_{i}}{\sum }g\left(z_{i}-z_{j}\right)\right)+\log\left(n\right). \end{array}$$

To learn a cross-domain representation to map the data of both domains to common space, we reduce the dependency of the domain indicator and the convolutional representation variables as much as possible. Since the mutual information measures the dependency, we minimize *I*(*π*; **z**) as follows:

(22) $$\begin{array}{c} \underset{W_{l}}{\text{min}}I\left(\pi ;z\right). \end{array}$$

In this way, we hope that the learned representations are independent from the domains as much as possible so that it can be generalized to adapt to both domains.

To construct the learning framework for the domain adaptation problem based on the classwise deep CNN representation model, we combine the objects of equations (8) and (22) for the minimization problem:

(23) $$\begin{array}{c} \underset{W_{l}}{\text{min}}\left\{o\left(W_{l}\right)=\underset{X_{i}\in X_{l}}{\sum }\left(\underset{X_{j}\in N_{i}^{+}}{\sum }A_{ij^{\prime }}^{+}{\left\|z_{i}-z_{j}\right\|}_{F}^{2}-\underset{X_{j}\in N_{i}^{-}}{\sum }A_{ij^{\prime }}^{-}{\left\|z_{i}-z_{j^{\prime }}\right\|}_{F}^{2}\right)\right.+C_{1}I\left(\pi ;z\right)+C_{2}{\left\|W\right\|}_{lF}^{2}\\ =\underset{X_{i}\in X_{l}}{\sum }\left(\underset{X_{j}\in N_{i}^{+}}{\sum }A_{ij^{\prime }}^{+}{\left\|z_{i}-z_{j^{\prime }}\right\|}_{F}^{2}-\underset{X_{j}\in N_{i}^{-}}{\sum }A_{ij^{\prime }}^{-}{\left\|z_{i}-z_{j^{\prime }}\right\|}_{F}^{2}\right)\\ +\frac{C_{1}}{nh_{i}}\underset{i:X_{i}\in X}{\sum }\left(\underset{j:X_{j}\in N_{i},\pi_{j}=\text{source}}{\sum }g\left(z_{i}-z_{j}\right)\right)\text{log}\left(\underset{j:X_{j}\in N_{i},\pi_{j}=\text{source}}{\sum }g\left(z_{i}-z_{j}\right)\right)\\ +\frac{C_{1}}{nh_{i}}\underset{i:X_{i}\in X}{\sum }\left(\underset{j:X_{j}\in N_{i},\pi_{j}=\text{target}}{\sum }g\left(z_{i}-z_{j}\right)\right)\text{log}\left(\underset{j:X_{j}\in N_{i},\pi_{j}=\text{target}}{\sum }g\left(z_{i}-z_{j}\right)\right)\\ \left.-\frac{C_{1}}{n}\underset{i:X_{i}\in X}{\sum }\text{log}\left(\left.\underset{j:X_{j}\in N_{i}}{\sum }g\left(z_{i}-z_{j}\right)\right)\right.+C_{2}{\left\|W_{l}\right\|}_{lF}^{2}\right\}, \end{array}$$

where ‖*W*_*l*_‖_*F*_^2^ term is used to control the complexity of the model to prevent the overfitting problem and *C*_1_ and *C*_2_ are the tradeoff parameters. In the objective, the first term is a large margin corresponding term, while the second and third terms are corresponding to the entropies of location distribution of convolutional representations over the neighborhood specified by source and target domains. The fourth them is corresponding to the entropy of the overall location distribution of representations.

### 2.3. Optimization

It is difficult to solve the problem of equation (23) because the classwise representations **z**_*i*_, *i*=1,…, *n* are the outputs of a deep CNN function, *f*_*l*_, while it also defines the neighborhoods and affinities. To solve the problem of equation (23), we treat the representation **z**_*i*_ as slack variables and introduce the following optimization problem:

(24) $$\begin{array}{c} \underset{W_{l},z_{1},\ldots ,z_{n}}{\text{min}}\left\{o\left(W_{l},z_{1},\ldots ,z_{n}\right)=\underset{X_{i}\in X_{l}}{\sum }\left(\underset{X_{j}\in N_{i}^{+}}{\sum }A_{ij^{\prime }}^{+}{\left\|z_{i}-z_{j}\right\|}_{F}^{2}-\underset{X_{j}\in N_{i}^{-}}{\sum }A_{ij^{\prime }}^{-}{\left\|z_{i}-z_{j^{\prime }}\right\|}_{F}^{2}\right)+\frac{C_{1}}{nh_{i}}\underset{i:X_{i}\in X}{\sum }\left(\underset{j:X_{j}\in N_{i},\pi_{j}=\text{source}}{\sum }g\left(z_{i}-z_{j}\right)\right)\text{log}\left(\underset{j:X_{j}\in N_{i},\pi_{j}=\text{source}}{\sum }g\left(z_{i}-z_{j}\right)\right)+\frac{C_{1}}{nh_{i}}\underset{i:X_{i}\in X}{\sum }\left(\underset{j:X_{j}\in N_{i},\pi_{j}=\text{target}}{\sum }g\left(z_{i}-z_{j}\right)\right)\text{log}\left(\underset{j:X_{j}\in N_{i},\pi_{j}=\text{target}}{\sum }g\left(z_{i}-z_{j}\right)\right)-\frac{C_{1}}{n}\underset{i:X_{i}\in X}{\sum }\text{log}\left(\underset{j:X_{j}\in N_{i}}{\sum }g\left(z_{i}-z_{j}\right)\right)+C_{2}{\left\|W_{l}\right\|}_{F}^{2}\right.\\ \text{s}.\text{t}.\;z_{i}=f_{l}\left(X_{i};W_{l}\right),\;\;i=1,\ldots ,n. \end{array}$$

To solve this problem, we use the ADMM algorithm. Following ADMM, we have the following optimization problem:

(25) $$\begin{array}{c} \underset{W_{l},z_{1},\ldots ,z_{n}}{\text{min}}\left\{o\left(W_{l},z_{1},\ldots ,z_{n}\right)+\frac{\rho }{2}\overset{n}{\underset{i=1}{\sum }}{\left|\left|f_{l}\left(X_{i};W_{l}\right)-z_{i}\right|\right|}_{F}^{2}+\overset{n}{\underset{i=1}{\sum }}\alpha_{i}^{⊤}\left(f_{l}\left(X_{i};W_{l}\right)-z_{i}\right)\right., \end{array}$$

where **α**_*i*_ is a dual variable for the constraint **z**_*i*_=*f*_*l*_(*X*_*i*_; *W*_*l*_) and *ρ* is its penalty parameter. We solve this problem by alternately updating the variables in an iterative algorithm.

#### 2.3.1. Updating of **z**_*i*_

The updating of **z**_*i*_ is conducted by solving the following ionization problem:

(26) $$\begin{array}{c} \underset{z_{i}}{\text{min}}\left\{o_{1}\left(z_{i}\right)=o\left(z_{i}\right)+\frac{\rho }{2}{\left\|f_{l}\left(X_{i};W_{l}\right)-z_{i}\right\|}_{F}^{2}+\alpha_{i}^{⊤}\left(f_{l}\left(X_{i};W_{l}\right)-z_{i}\right)\right., \end{array}$$

and we solve it by a gradient descent method:

(27) $$\begin{array}{c} z_{i}\;⟵\;z_{i}-\rho \nabla_{z_{i}}o_{1}\left(z_{i}\right), \end{array}$$

where *ρ* is a descent step parameter and ∇_**z**_*i*__*o*_1_(**z**_*i*_) is the gradient function regarding **z**_*i*_,

(28) $$\begin{array}{c} \nabla_{z_{i}}o_{1}\left(z_{i}\right)=\nabla_{z_{i}}o\left(z_{i}\right)-\rho \left(f_{l}\left(X_{i};W_{l}\right)-z_{i}\right)-\alpha_{i}. \end{array}$$

#### 2.3.2. Updating of *W*_*l*_

Updating of *W*_*l*_ is conducted by solving the following minimization problem:

(29) $$\begin{array}{c} \underset{W_{l}}{\text{min}}\left\{o_{2}\left(W_{l}\right)=C_{1}{\left\|W_{l}\right\|}_{F}^{2}+\frac{\rho }{2}{\left\|f_{l}\left(X_{i};W_{l}\right)-z_{i}\right\|}_{F}^{2}+\alpha_{i}^{⊤}\left(f_{l}\left(X_{i};W_{l}\right)-z_{i}\right)\right.. \end{array}$$

We also use the backprorogation algorithm to solve this problem, based on the chain rule:

(30) $$\begin{array}{c} W_{l}\;⟵\;W_{l}-\rho \nabla_{W_{l}}o_{2}\left(W_{l}\right),\\ \nabla_{W_{l}}o_{2}\left(W_{l}\right)=2C_{1}W_{l}+\rho {\left(f_{l}\left(X_{i};W_{l}\right)-z_{i}\right)}^{⊤}\nabla_{W_{l}}f_{l}\left(X_{i};W_{l}\right)+\alpha_{i}^{⊤}\nabla_{W_{l}}f_{l}\left(X_{i};W_{l}\right). \end{array}$$

#### 2.3.3. Updating of *α*_*i*_

The dual variable is updated by gradient ascent:

(31) $$\begin{array}{c} \alpha_{i}\;⟵\;\alpha_{i}+\rho f_{l}\left(X_{i};W_{l}\right). \end{array}$$

### 2.4. Overall Learning Algorithm of MMITR

In this section, we give the overall iterative learning algorithm of the proposed minimum mutual information transfer representation (MMITR) method. In this algorithm, it has an updating strategy similar to the expectation-maximization (EM) algorithm. In each iteration, we firstly fix domain transfer representations to update the inter- and intraclass affinity metrics in an E-step and then fix the inter- and intraclass affinity metrics to update the CNN parameters and the representations in an M-step. The iterations are stopped until a maximum iteration number is reached or the objective value reaches a threshold. The overall algorithm is described in Algorithm 1.

### 2.5. Prediction of a New Data Point

When we have a new data point, *X*, to classify it, we calculate its classwise representation and corresponding margin regarding each class:

(32) $$\begin{array}{c} z=f_{l}\left(X;W_{l}\right),\\ m_{l}\left(X\right)=\underset{X_{j}\in N_{X}^{-}}{\sum }A_{ij^{\prime }}^{-}{\left\|z_{i}-z_{j^{\prime }}\right\|}_{F}^{2}-\underset{X_{j}\in X_{X}^{+}}{\sum }A_{ij^{\prime }}^{+}{\left\|z_{i}-z_{j^{\prime }}\right\|}_{F}^{2},\\ l=1,\ldots ,L, \end{array}$$

where the intra- and interclass neighborhood and affinity are calculated according to the classwise representations. The new data point is assigned to a class which gives the maximum margin:

(33) $$\begin{array}{c} y^{*}=\arg\;\;\max_{l=1}^{L}m_{l}\left(X\right). \end{array}$$

## 3. Experiments

### 3.1. Datasets

In our experiments, we use the following datasets as benchmark datasets:

  Office-31: this dataset has 4,652 images of 31 classes. The images are from three different domains: Amazon (images downloaded from http://www.amazon.com), Webcam (photos taken by web camera), and DSLR (photos taken by digital SLR camera).
  ImageCLEF-DA: this dataset is composed of images of 12 classes of four domains. Each domain is a unique database, including Caltech-256, ImageNet ILSVRC 2012, Pascal VOC 2012, and Bing.
  Email spam: this dataset has email texts of spam and nonspam. The data are collected from three users, and each user has 2,500 emails. Each user is treated as a domain.
  Extended Cohn-Kanade (CK+): this dataset is an image set for facial expression recognition. It has images of 123 subjects, and each subject is treated as a domain. This dataset has 593 videos in total, and for each video, there are about 20 frames. Each image of a face belongs to one of the 7 expression classes.
  Amazon: this dataset is a text classification task dataset. The texts are from three different domains, and each domain is the review for products of books, DVD, and music. For each domain, there are 2,000 positive review texts and 2,000 negative review texts.

### 3.2. Experimental Setting

In this experiment, we use each domain of a dataset as a target domain in turn and the remaining domains as the source domains. For each test domain, we use the leave-one-out protocol to split it into a training set and a test set. Each data point of a target domain is used as a test data point in turn, and the remaining data points are combined to form a training set. The training set of the target domain is randomly split to an unlabeled set and a labeled set, with equal size. The data points of the source domains are always treated as labeled in our setting. Our algorithm is performed over the training set to learn the parameters of the classwise representation model and the domain-independent representations and then used to classify the test data points. The classification accuracy is used to evaluate the performance of the algorithm.

### 3.3. Results

In our experiment, we first study the properties of the algorithm experimentally, including its sensitivity to the tradeoff parameters and its convergence property to iteration numbers. Then, we compare its performance to state-of-the-art domain transfer learning algorithms.

#### 3.3.1. Algorithm Property Evaluation

**Figure 2:**
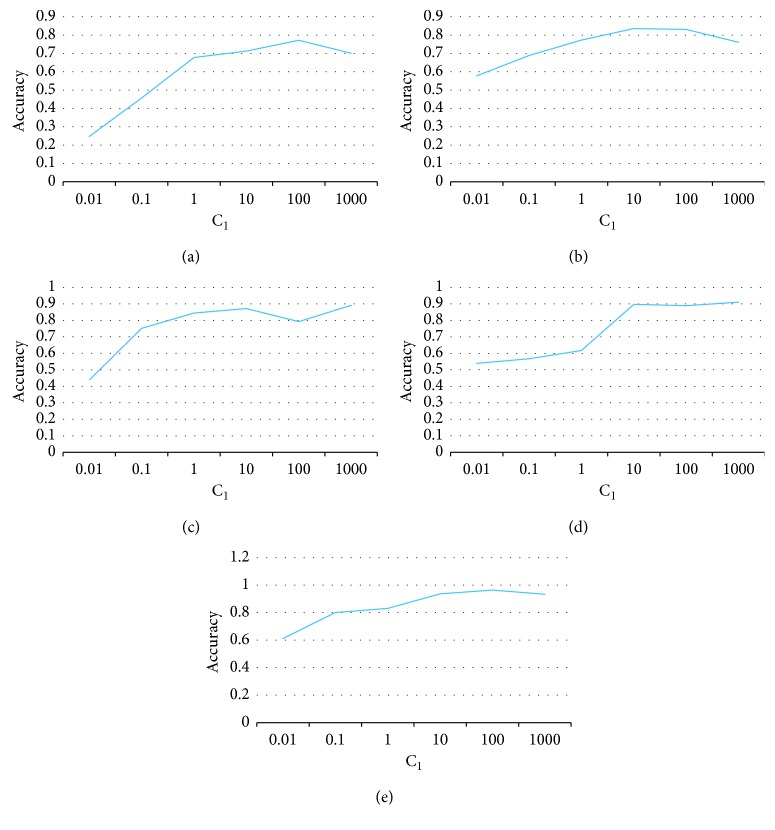
Tradeoff parameter sensitivity study of *C*_1_. (a) Office-31. (b) ImageCLEF-DA. (c) Email spam. (d) CK+. (e) Amazon.

*(1) Sensitive to Tradeoff Parameters*. There are two tradeoff parameters in our algorithm: *C*_1_ and *C*_2_. They are the weights of the mutual information term and the complexity reduction term in our object. We plot the accuracy of our algorithm regarding different values of *C*_1_, as shown in Figure 2. From this figure, we observe that the accuracy improves in most cases when the value of *C*_1_ increases. Since *C*_1_ is the weight of the mutual information term to measure how dependent the representation is from the domain, this indicates that a more domain-independent representation helps the classification in the target domain. Actually, the more independent the representation is from the domain, the better the data of different domains are merged. Thus, the source domain can benefit the learning problem in the target domain more. This phenomenon is even more obvious in the CK + dataset; when *C*_1_ grows from 1 to 10, the accuracy is boosted significantly. This is a piece of strong evidence how the minimum mutual information improves the transfer learning.

**Figure 3:**
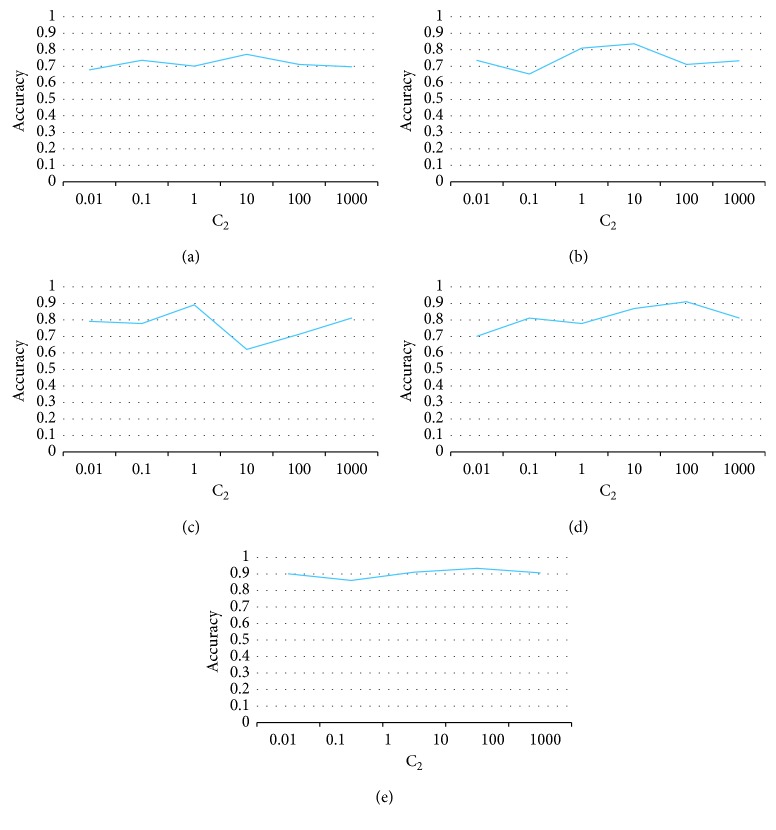
Tradeoff parameter sensitivity study of *C*_2_. (a) Office-31. (b) ImageCLEF-DA. (c) Email spam. (d) CK+. (e) Amazon.

The accuracy curves of classification with different values of *C*_2_ are shown in Figure 3. From this figure, we can see that the proposed algorithm is stable to the change of *C*_2_. Since the algorithm is not sensitive to the change of *C*_2_, the tuning of this parameter will be easy for a specific dataset. One only exception is the case when *C*_2_ varies between 1 and 10, the accuracy changes dramatically.

**Figure 4:**
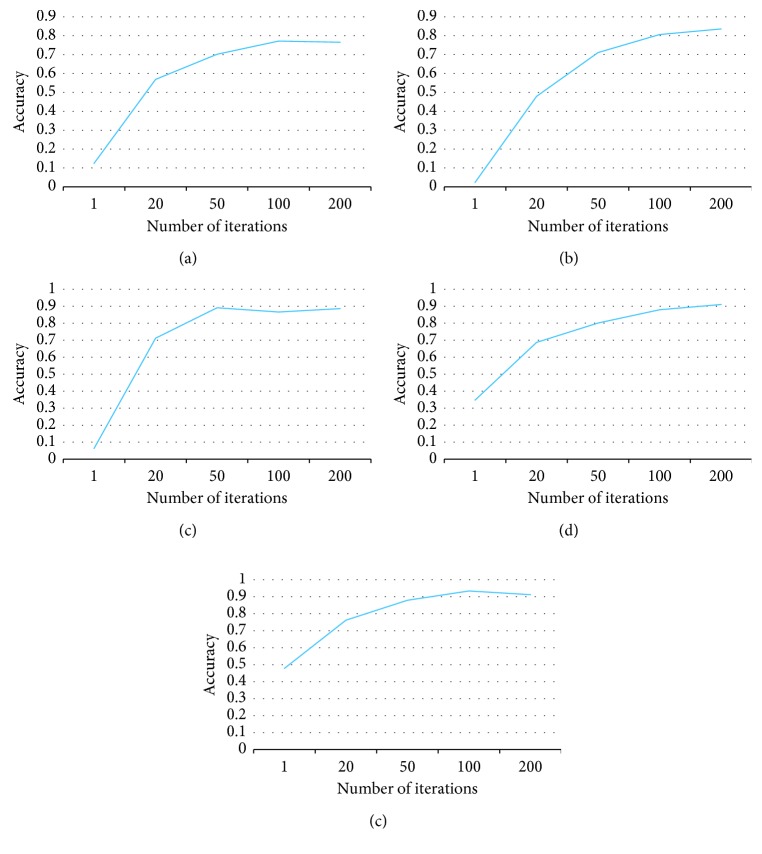
Convergence analysis. (a) Office-31. (b) ImageCLEF-DA. (c) Email spam. (d) CK+. (e) Amazon.

*(2) Convergence Analysis*. Since our algorithm is an iterative algorithm, it is critical to know when to stop the iterations. We study the convergence of the algorithm by plotting the accuracy over different datasets with varying numbers of iterations in Figure 4. According to the curves in the figure, in most datasets, the algorithm gives a better accuracy when the iteration number grows and then becomes stable after about 100 iterations. For the email spam dataset, the algorithm converges at 50 iterations.

**Remark 4 .**

**Figure 5:**
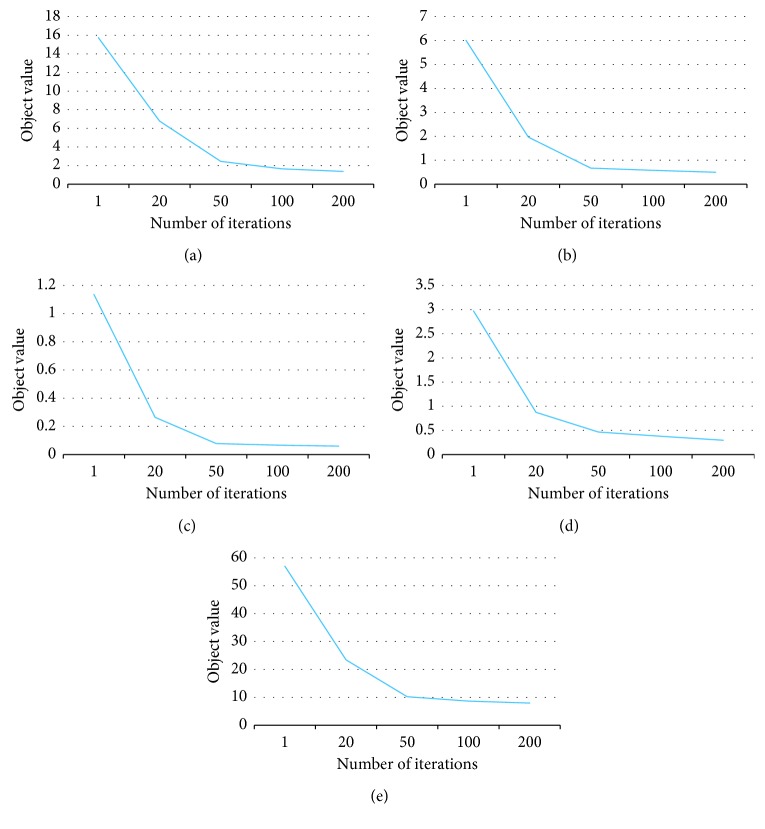
Objective value curve of ADMM algorithm. (a) Office-31. (b) ImageCLEF-DA. (c) Email spam. (d) CK+. (e) Amazon.

To solve a minimization problem in our algorithm, we employed the ADMM algorithm. To verify if the ADMM algorithm solves the optimization of the minimization problem effectively, we plot the object values of the learning problem with increasing numbers of iterations in Figure 5. As we can see from the curves, the object value decreases stably as the number of iterations increases, until it reaches a convergency, and then the changing of object values becomes small. This is a strong evidence that ADMM algorithm solves the optimization problems effectively (Table 1).

#### 3.3.2. Comparison to State of the Arts

We compare our algorithm, MMITR, to several state-of-the-art transfer learning algorithms, including the Deep Adaptation Network (DAN) [4], Selective Transfer Machine (STM) [1], Semisupervised Kernel Matching Domain Adaptation (SSKMDA) [6], and Domain Transfer Multiple Kernel Learning (DTMKL) [2]. In Table 2, we have provided a detailed list of algorithms compared in the experiment, regarding the aspect of data representation components and domain matching criteria.

**Figure 6:**
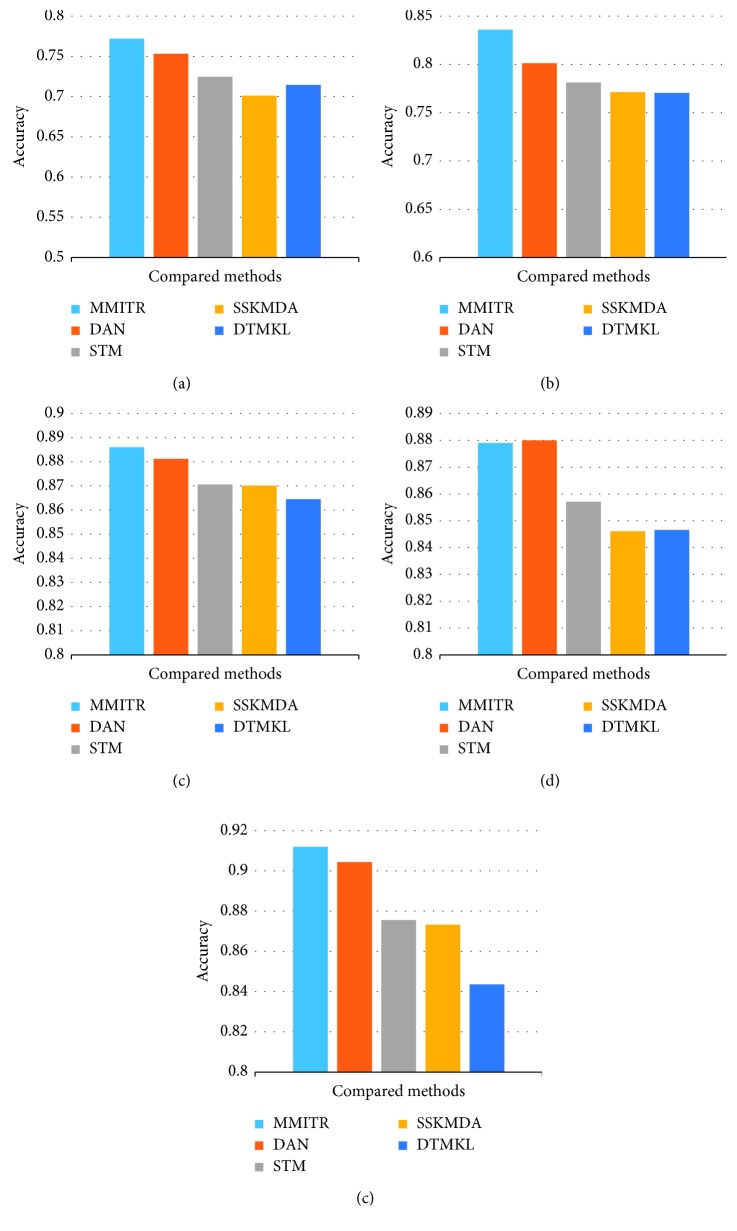
Comparison of accuracy of state of the arts. (a) Office-31. (b) ImageCLEF-DA. (c) Email spam. (d) CK+. (e) Amazon.

The comparison of accuracy results is given in Figure 6. In this figure, we can observe that the proposed method outperforms the other methods in four experiments out of five. In experiments over three datasets (Office-31, ImageCLEF-DA, and Amazon), our algorithm outputs the second best method, DAN, by a large margin. For the dataset of CK+, DAN outperforms our method by a slight amount. Both DAN and our method MMITR are based on deep learning model, but our method tries to learn domain-independent deep representations, while DAN tries to learn a deep learning model to represent the data points so that mean of representations of source domain and target domain can be similar to each other. According to the results, MMITR outperforms DAN in most cases; we conclude that domain-independent deep representation is more suitable for domain transfer learning than domain-mean matched representation. The other methods are also based on mean-matching of domain transfer representations, but using a shallow model instead of a deep model; thus, they are not able to explore hierarchal deep features. This again verifies the effectiveness of the deep model.

**Remark 5 .**

The conditions of the methods of the results reported in 6 are described in details as follows. For the DAN algorithm, it has two hyperparameters: the MMD Penalty *λ* and the Entropy Penalty *γ*, and we set their values to 1 and 0.1, respectively. For the STM algorithm, there are two hyperparameters: *C* for the tradeoff between maximal margin and training loss and *λ* for the tradeoff between the SVM empirical risk and the domain mismatch loss. In this experiment, we set both their values to 1. For the SSKMDA algorithm, it has five tradeoff parameters between the model components: *μ*, *η*, *β*, *γ*_*s*_, *γ*_*t*_, and their value setting in our experiment are 10, 2, 0.1, 0.1, and 1, respectively. The DTMKL algorithm has only one hyperparameter: the regularization parameter *C*, and we set it to 0.5 in the experiments. For our algorithm MMITR, it has two tradeoff parameters, *C*_1_ and *C*_2_; for each benchmark dataset, we report the best results among the results obtained by using different values of *C*_1_ and *C*_2_.

## 4. Conclusions and Future Works

In this paper, we proposed a novel framework for transfer learning. Not like the traditional transfer learning which tries to match the representations of source domain and target domain, we proposed to learn domain-independent representations. We argue to measure the dependency of learned deep representations and domain by mutual information and learn the domain-independent deep representations by minimizing the mutual information. We also proposed a practical estimation method for the mutual information between domain and deep representations. A classwise deep representation neural network work is trained under this framework and used to classify new data points. Experiments over benchmark datasets for transfer learning verify the effectiveness of the proposed method.

**Remark 6 .**

The new concept proposed in this paper is a novel domain transfer learning framework which minimizes the mutual information between the domain transfer representations and the domain indicators so that the gaps among domains can be effectively leveraged and a common representation space is learned. The new method developed in this is a novel iterative learning algorithm to learn the domain transfer representations based on CNN models.

## Figures and Tables

**Algorithm 1:**
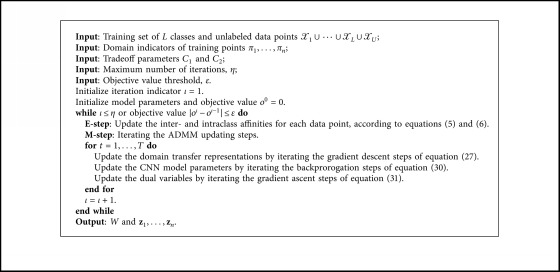
Iterative learning algorithm of MMITR.

**Table 1:** Definitions of symbols.

Symbol	Definition
*X*_*i*_	*i*-th data point
*𝒳*_*l*_	Set of data points of the *l*-th class
*𝒳*_*U*_	Set of unlabeled data points
**z**	Domain transfer representation of a data point
*W*_*l*_	Parameters of the CNN model of the *l*-th class
*N*_*i*_^+^	Intraclass neighborhood of the *i*-th data point
*N*_*i*_^−^	Interclass neighborhood of the *i*-th data point
*A*_*ij*_^+^	Intraclass affinity between the *i*-th and *j*-th data points
*A*_*ij*′_^+^	Interclass affinity between the *i*-th and *j*′-th data points
*π*_*i*_	Domain indicator of the *i*-th data point
*ω*_*ij*_	Soft weight of the the *j*-th data point in the *i*-th neighborhood

**Table 2:** Compared methods.

Method	Data representation	Domain matching criterion
MMITR	CNN	Mutual information minimization
DAN	CNN	Maximum mean discrepancy (MMD)
STM	—	Kernel mean-matching (KMM)
SSKMDA	Multikernel learning	Hilbert schmidt independence criterion (HSIC)
DTMKL	Multikernel learning	MMD

## Data Availability

All the datasets used in this paper are publicly accessed.
